# Beta wave enhancement neurofeedback improves cognitive functions in patients with mild cognitive impairment: A preliminary pilot study

**DOI:** 10.1097/MD.0000000000018357

**Published:** 2019-12-16

**Authors:** Jung-Hee Jang, Jieun Kim, Gunhyuk Park, Haesook Kim, Eun-Sun Jung, Ji-yun Cha, Chan-young Kim, Siyeon Kim, Jun-Hwan Lee, Horyong Yoo

**Affiliations:** a Department of Neurologic Disorders & Aging Brain Constitution, Dunsan Korean Medicine Hospital, Daejeon University; b Clinical Medicine Division, Korea Institute of Oriental Medicine; c Herbal Medicine Resources Research Center, Korea Institute of Oriental Medicine, Naju-si, Jeollanam-do, Republic of Korea.

**Keywords:** functional near-infrared spectroscopy, mild cognitive impairment, neurofeedback

## Abstract

**Background:**

Mild cognitive impairment (MCI) is a symptom characterizing cognitive decline and a transitional state between normal aging and dementia; however, there is no definitive diagnosis and treatment for MCI. Neurofeedback (NF), which is a training mechanism that employs operant conditioning to regulate brain activity, has been increasingly investigated concerning its beneficial effects for dementia and MCI.

**Methods:**

This study investigated cognitive improvement and hemodynamic changes in the prefrontal cortex (PFC) following NF training in patients with MCI. Five patients with MCI received NF training for enhanced beta band activity in the dorsolateral PFC—16 sessions for 8 weeks—with each session divided into 9 5-minute trials. The primary outcome measure was a cognitive assessment tool: the Korean version of the Montreal Cognitive Assessment. The secondary outcome measures were the Central Nervous System Vital Signs for neurocognitive testing, hemodynamic changes using functional near-infrared spectroscopy in the PFC during a working-memory task, and Beck Depression Inventory scores.

**Results:**

After completing the training, patients’ cognitive function significantly improved in domains such as composite memory, cognitive flexibility, complex attention, reaction time, and executive function. Increased electroencephalogram beta power was observed over NF training sessions (Spearman rank correlation test: *r* = 0.746, *P* = .001). The threshold value for gaining positive feedback from pre-NF baseline on beta power significantly increased (Spearman rank correlation test: *r* = 0.805, *P* = .001). Hemodynamic response in PFC changed after NF training, and individual differences were identified. Specifically, hypoactivation of the hemodynamic response by emotional distraction recovered following NF training.

**Conclusion:**

We suggest that patients’ cognitive processing efficiency was improved by the NF training. These beneficial results suggest that NF training may have potential therapeutic applications to prevent the progression from MCI to dementia.

Trial registration number: Clinical Research Information Service (KCT0003433).

## Abstract 2

Supplemental Digital Content is available in the text

## 1 Introduction

Dementia is a neurodegenerative disease characterized by progressive deterioration in cognitive function and the capacity for daily function; problematically, dementia is associated with enormous costs worldwide^[1]^ as its prevalence is expanding rapidly.^[2]^ Therefore, it is important to research diagnostic and preventative approaches that may be used before the onset of dementia.

Mild cognitive impairment (MCI) refers to an intermediate clinical state between the cognitive changes in normal aging and early dementia.^[3–6]^ MCI is associated with the earliest manifestation of Alzheimer disease (AD) and may develop into dementia.^[6–10]^ Individuals with MCI do not experience interference with their daily lives; however, they display cognitive decline over time.^[6,11]^ The management of MCI to prevent dementia is important. Cognitive function is characterized across 5 domains: learning and memory, language, visuo-spatial perception, execution, and psychomotor behavior. If only one domain is impaired, individuals are diagnosed with MCI. The assessment for the diagnosis of MCI can be obtained from patients’ medical history, a mental status examination, or neuropsychological testing^[6]^; however, there is no definitive standard for MCI diagnosis and treatment.^[6,12]^

To develop a diagnostic tool for MCI, many researchers have searched for a biomarker for MCI using cerebrospinal fluid, electroencephalogram (EEG), magnetic resonance imaging, and positron emission tomography.^[11]^ In addition, studies addressing therapies for MCI have been performed; however, there are no approved pharmacological treatments for MCI.^[6,12]^ Notably, there is some evidence that several non-pharmacological treatments may be neuroprotective in improving cognitive impairment, including cognitive training^[11,13,14]^ and physical exercise.^[15]^

Neurofeedback (NF) is a training mechanism that employs operant conditioning to reward or inhibit desirable and undesirable brain activity, respectively.^[13]^ Its beneficial effects have been mainly reported concerning attention deficit hyperactivity disorder,^[16]^ epilepsy,^[17]^ autism,^[18]^ depression,^[19]^ and anxiety.^[20]^ In addition, studies have reported the effectiveness of NF training in improving cognitive function of patients with dementia.^[13,21]^ After NF training in patients with dementia, patients’ scores increased on the Mini Mental Status Examination, which is the most widely used test for cognitive impairment. Additionally, an EEG indicated reduced theta rhythm and activation of alpha and beta rhythms following NF training.^[13,21]^ Recently, several studies demonstrated improved cognitive function using NF training in patients with MCI.^[11,22,23]^

As neuroimaging technology, functional near-infrared spectroscopy (fNIRS) is a non-invasive optical method comprising monitoring oxygenation and its kinetics in the cerebral cortex.^[24]^ fNIRS uses near-infrared light to measure oxygenated hemoglobin (oxy-Hb), deoxygenated hemoglobin (deoxy-Hb), and total hemoglobin. The concentrate/ion of oxy-Hb is an indicator of regional cerebral blood flow (CBF) changes,^[25]^ and oxy-Hb changes are equivalent to cortical activation corresponding to CBF in functional magnetic resonance imagery (fMRI) research.^[26]^ The advantages of fNIRS are portability, low cost, and relative insensitivity to movement^[27]^; therefore, it is suitable as a diagnostic tool for early diagnosis of cognitive impairment.^[25]^ In a previous fNIRS study, patients with MCI showed reduced brain activity in frontal and temporal cortices during a working memory (WM) task (0- and 1-back tasks) compared with healthy controls—thus confirming the presence of functional deficits.^[28]^ Another fNIRS study used a semantic verbal fluency task to assess activity in the prefrontal cortex (PFC) of patients with MCI. The hemodynamic response manifested hyperactivation in MCI compared with healthy controls and individuals with AD, suggesting the presence of a compensatory mechanism in patients with MCI.^[27]^ These conflicting results may be attributed to differences in tasks and brain regions of interest. Therefore, it is necessary to investigate changes in brain activity in MCI using various tasks.

In the present study, hemodynamic responses in the PFC of patients with MCI following NF were assessed during a WM task using the delayed match-to-sample (DMTS) task, including the emotional distraction phase—consisting of highly arousing pictures—as a WM task. Emotion is known to influence various aspects of cognition by enhancing or hindering them.^[29]^ We hypothesized that PFC activity would be altered by emotional distraction during the WM task and regulated by NF training.

In this study, we assessed cognitive improvement with the Korean version of the Montreal Cognitive Assessment (MoCA-K), the Central Nervous System Vital Signs (CNSVS), and brain activity changes in the PFC following NF training in patients with MCI. We investigated the hemodynamic responses to emotional distraction in patients with MCI and whether altered brain responses were regulated by NF training. Hemodynamic changes following NF training have not been examined, and the results provide novel insights into the brain activity of individuals with MCI.

## 2 Methods

### 2.1 Study design and setting

A small, open-label preliminary pilot trial was performed at the Dunsan Korean Medicine Hospital of Daejeon University in South Korea beginning in July 2018. Participants received 45 minutes of NF training twice a week for 8 weeks. The primary outcome measure was MoCA-K scores, and the secondary outcome measures were the CNSVS, hemodynamic changes in the PFC using fNIRS, and Beck Depression Inventory (BDI) scores. The study setting and site of data collection was the Clinical Trial Center at Dunsan Korean Medical Hospital, Daejeon University, South Korea.

### 2.2 Participants and recruitment

Five participants with MCI were recruited through banners placed in the community and hospitals from July to November 2018. Participants who fit the eligibility criteria provided written, informed consent after a consultation, and received information regarding this preliminary pilot study.

#### 2.2.1 Inclusion criteria

Inclusion criteria consisted of the following: aged 40 to 80 years; understanding the study objective and providing informed consent; at least 6 years of education; meeting the Peterson diagnostic criteria for MCI (complaint of defective memory, normal activities of daily living, normal general cognitive function, abnormal memory function for age, and absence of dementia),^[30]^ with memory loss for at least 3 months; a Hachinski ischemic score ≤6 (to exclude patients with vascular dementia); and an MoCA-K score ≤22.^[31]^

#### 2.2.2 Exclusion criteria

Exclusion criteria consisted of the following: dementia according to the fourth edition of the Diagnostic and Statistical Manual of Mental Disorders; a history of neurological diseases that can cause cognitive impairment, such as Parkinson disease, stroke, cerebral hemorrhage, tumors, or normal pressure hydrocephalus; having received any treatment for MCI within the previous 2 weeks; having participated in other clinical trials within the last 4 weeks; ineligibility to participate in this clinical study per the coordinator's discretion or exhibiting a non-cooperative attitude; and inability to complete the fNIRS measurements for any reason.

### 2.3 Interventions

#### 2.3.1 NF training

Patients underwent 16 sessions of NF training (twice a week for 8 weeks); each session comprised 9 5-minute trials. A single-channel EEG signal in the dorsolateral PFC (dlPFC, at F6 using 10–20 EEG electrode placement system) was collected and beta band power (12–15 Hz, low beta waves) was calculated to feed to patients. Patients were instructed to enhance beta wave power in dlPFC based on the following. Previous studies showed that patients with MCI presented decreased beta power at rest and less task-evoked beta responses during a task.^[32,33]^ The dlPFC (Brodmann area [BA] 46) is known to play a role in executive functions including WM. Several studies with magnetoencephalography and fMRI reported that the dlPFC is involved in various WM processes.^[34–37]^

Further, a meta-analysis found that activation to the nonverbal DMTS task, which we similarly adopted in our study, extended to the dlPFC (BA 46).^[38]^ An EEG signal was acquired using an NF system (ProComp Infiniti System, Thought Technology Ltd., Quebec, Canada) with a sampling rate of 256 Hz. The active electrode was placed on the scalp over the right dlPFC. The reference electrode was placed on the left earlobe, which is located on the side opposite to the active electrode; and the ground electrode was placed on the right earlobe. The electrode locations were cleaned with an abrasive conductive gel (Nuprep, Weaver and company, Colorado), and 3 electrode cups were filled with a conductive gel (Ten 20 conductive paste, Weaver and company, Colorado) and electrode impedance was kept below 5 kΩ. To calculate the amplitude of beta power individually, participants were asked to sit comfortably with their eyes open and look ahead without visual stimulation for 1 minute. Once the threshold was set, participants used their own strategy to increase beta band power by moving a boat to the other side or changing blurred flowers vivid on a computer screen. In sum, individual threshold was determined based on mean beta band power from pre-NF baseline (1 minute) for each session. Mean beta power from each trial (5 minutes) was calculated after removing physical artifacts (eye-blinks) from EEG data; then, they were averaged across trials. We investigated dose-dependent changes in beta power from pre-NF baseline and NF session.

### 2.4 Outcome measures

#### 2.4.1 Primary outcome—MoCA-K

MoCA-K scores^[39]^ were the primary outcome in this study. We used the validated Korean version of the MoCA (MoCA-K) as a cognitive assessment tool.^[31,39]^ The MoCA-K comprises several subscales: visuospatial ability, executive function, attention-concentration-WM, language, short-term memory recall, and orientation to time and place to evaluate individuals’ overall cognitive function. The measure employs a 30-point scale; the cutoff score for detecting MCI is 22/23.^[31]^ Scores were evaluated at baseline (before NF training), 4 weeks after NF training (session 8), and 8 weeks after NF training (session 16).

#### 2.4.2 Secondary outcomes

The secondary outcomes comprised the CNSVS for neurocognitive testing; hemodynamic changes in the PFC during the WM task; and BDI scores, which are associated with cognitive dysfunction.

##### 2.4.2.1 CNSVS

The CNSVS is a computerized neurocognitive test battery that is known to be sensitive for distinguishing MCI.^[40,41]^ It comprises 7 tests: verbal and visual memory, finger tapping, symbol digit coding, Stroop, shifting attention, and continuous performance testing.^[42,43]^ Responses refer to specific domains such as composite memory, processing speed, psychomotor speed, executive function, reaction time, complex attention, and cognitive flexibility.^[42]^ The computerized neurocognitive assessment software was purchased and analyzed in Korea.

##### 2.4.2.2 The fNIRS data collection and WM task

The fNIRS data (NIRSIT, OBELAB, Inc., Seoul, Korea) in the PFC were collected before and after the NF training during the WM task. This system utilizes the transmission of near-infrared light at 2 wavelengths (780 and 850 nm), with a power of 1 mW per wavelength, and comprises 24 light-emitters and 32 photo-detectors. A sampling rate of 8.138 Hz was used to acquire the fNIRS data. The probes were placed above the prefrontal lobe of each participant. The fNIRS data from the PFC were acquired when participants performed a WM task. Data collection and analysis were performed using NIRSIT PC tool version 2.4 and NIRSIT Analysis Tool version 2.2 (NIRSIT, OBELAB, Inc., Seoul, Korea), respectively. Based on the difference in absorption spectra of the detected signal, assuming constant light scattering, changes in the concentration of oxy-Hb in the targeted PFC region were measured using the modified Beer-Lambert law. The data for the measured oxy-Hb were exported into .csv file format and analyzed using NIRSIT Analysis tool version 2.2 developed using MATLAB (MATLAB and Statistics Toolbox Release 2012b, The Math Works, Inc., Natick, MA); thus, we calculated the mean oxy-Hb concentration for each patient.

**Figure 1:**
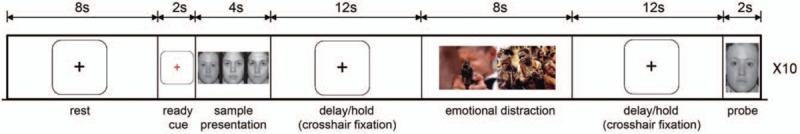
Scheme showing the details of the working memory task.

The DMTS task is a widely used procedure when studying WM. The DMTS task is composed of 3 phases: sample presentation (stimulus is presented and participants memorize the sample), delay/hold (no stimulus and maintain a representation of the sample), and probe (making a decision based on memory of the sample).^[38]^ In the present study, we revised the DMTS task with emotional distraction based on Dolcos and McCarthy's study^[44]^ (Fig. 1). Three emotionless faces were followed by highly arousing pictures from the International Affective Picture System^[45]^ (mean arousal and valence ratings were 6.4 and 1.8 as emotional distraction). Participants were instructed to press the button (O/X button) depending on whether the face at the probe existed in the sample presentation period or not. To characterize hemodynamic changes associated with NF training, using fNIRS during the DMTS task, mean oxy-Hb values were analyzed.

##### 2.4.2.3 BDI

The BDI was used to assess depression, which is a well-known risk factor for MCI.^[46]^ The BDI is one of the most widely used self-report measures of depression. It assesses emotional, behavioral, and somatic symptoms associated with depression.^[47]^

### 2.5 Safety assessment and adverse events

A safety assessment for all participants was performed via a clinical laboratory examination at baseline and after completion of NF training. Adverse events were defined as undesirable and/or unexpected medical findings. Participants were examined for any adverse reactions at every visit; no such effects were reported.

### 2.6 Statistical analyses

This was a small open-labeled preliminary study, and parametric statistical analyses were not suitable. The Friedman test was performed to test the difference in MoCA-K scores from baseline, after 8 and 16 sessions of NF training. We also performed post-hoc tests using Wilcoxon signed-rank tests to compare the MoCA-K scores at baseline with the period after 8 and 16 sessions of NF training with a Bonferroni correction, resulting in a significance level set at *P* < .025. For CNSVS and BDI scores, Wilcoxon signed-rank tests were used to assess the difference between the scores before and after the NF training. Spearman rank correlation tests were performed between EEG beta power and number of NF training sessions. Statistical significance was set at *P* < 0.05. Statistical analyses were performed using SPSS for Windows, version 24.0, (Chicago, IL).

### 2.7 Ethical considerations and study registration

The study protocol was approved by the Research Ethics Committee of Dunsan Korean Medicine Hospital, Daejeon University, and they were responsible for supervising all aspects of the study. All participants were willing to participate. The study protocol (version 1.2, 2018–08–31) was registered with the Clinical Research Information Service of the Republic of Korea (KCT0003433).

## 3 Results

### 3.1 Demographic and clinical characterization

Participants’ demographic characteristics and baseline results of the cognitive assessments are shown in Table 1.

**Table 1:**
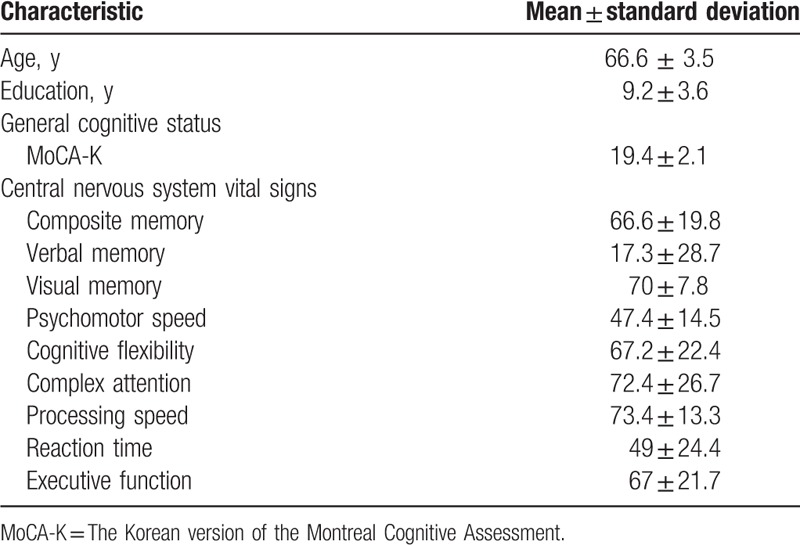
Participants’ demographics and cognitive characteristics.

### 3.2 Cognitive assessment

**Figure 2:**
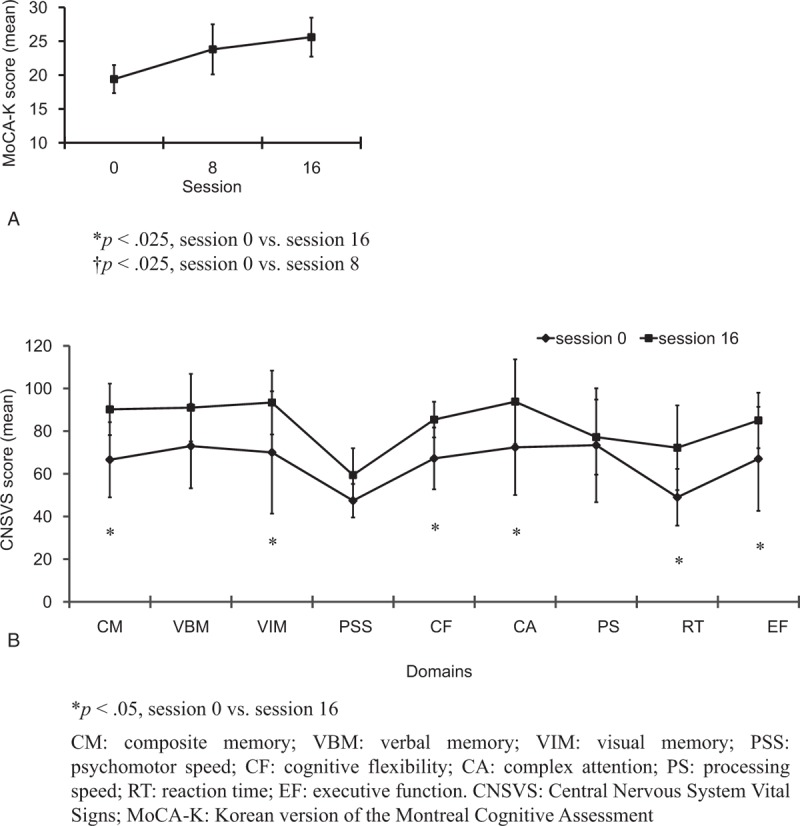
MoCA-K and CNSVS scores (mean ± SD) for 5 participants with mild cognitive impairment. MoCA-K scores were assessed at baseline and post-NF training (sessions 8 and 16). CNSVS scores were assessed at baseline and following NF training (session 16). MoCA-K scores were significantly different (Friedman test, *χ*^2^(2) = 8.32, *P* = .016). MoCA-K scores at sessions 8 and 16 were not significantly different compared with those at baseline (Wilcoxon signed-rank tests with a Bonferroni correction; ^∗^*P* < .025, session 0 versus session 16; ^†^*P* < .025, session 0 versus session 8). Wilcoxon signed-rank tests were used for CNSVS scores (∗*P* < .05, session 0 versus session 16). Error bars represent SD. CNSVS = Central Nervous System Vital Signs; MoCA-K = the Korean version of the Montreal Cognitive Assessment; NF = neurofeedback; SD = standard deviation.

The cognitive assessment results (MoCA-K and CNSVS) are illustrated in Fig. 2. The individual data concerning clinical outcomes before and after NF training are summarized in Table 2. There was a statistically significant difference in MoCA-K scores, χ^2^(2) = 8.32, *P* = .016. Post-hoc testing with Wilcoxon signed-rank tests was conducted with a Bonferroni correction, resulting in a significance level set at *P* < .025. Compared with baseline, there were no statistically significant differences in the period after 8 sessions of NF (*Z* = –2.03, *P* = .042), nor after 16 sessions of NF (*Z* = –2.03, *P* = .042), (baseline: 19.4 ± 2.07; after 8 sessions of NF: 23.8 ± 3.70; after 16 sessions of NF: 25.6 ± 2.88).

**Table 2:**
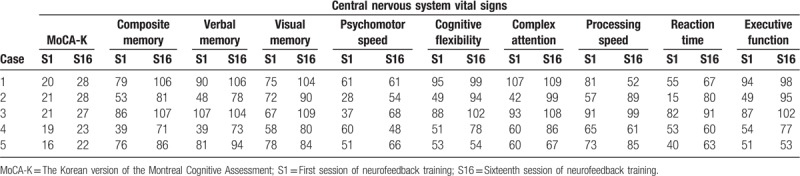
Individual data on clinical outcomes.

Concerning the CNSVS, scores for each domain were classified into 5 categories: very low (<2nd percentile), low (2nd to 8th percentile), low average (9–24th percentile), average (25–74th percentile), and above average (greater than 74th percentile). Before NF training, composite memory, psychomotor speed, cognitive flexibility, reaction time, and executive function scores were very low, and complex attention and processing speed scores were low. After completion of NF training, participants either improved or remained unchanged as follows: composite memory showed significant improvement from very low to average (*P* = .043), cognitive flexibility (*P* = .043), and executive function (*P* = .043) improved from very low to low average, complex attention improved from low to average (*P* = .043), reaction time slightly improved from very low to low (*P* = .043), and psychomotor speed (*P* = .144) and processing speed (*P* = .50) did not change.

### 3.3 EEG results

**Figure 3:**
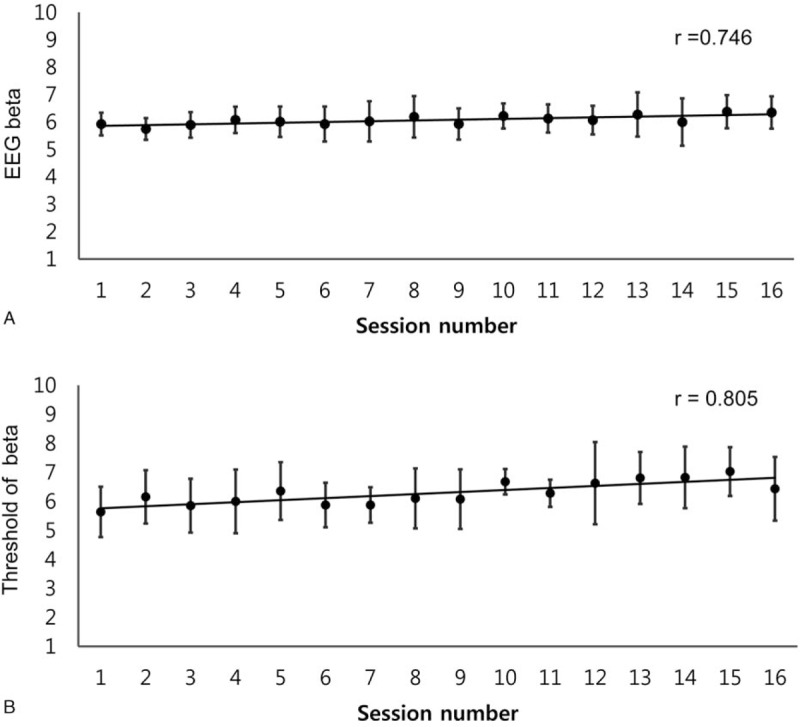
Correlations between number of NF training sessions and beta band activity for 5 participants with mild cognitive impairment (Spearman rank correlation test: *r* = 0.746, *P* = .001, A). A strong correlation between the threshold value for gaining positive feedback on beta band and NF training session was found (Spearman rank correlation test: *r* = 0.805, *P* = .001, B). EEG = electroencephalogram; NF = neurofeedback.

Mean EEG beta power was calculated using EEG signal during NF trials. It significantly increased throughout the NF training sessions (Spearman rank correlation test: *r* = 0.746, *P* = .001; Fig. 3A). We also tested the threshold value for gaining positive feedback for each session and found that beta power from pre-NF baseline significantly increased in a dose-dependent manner following NF training (Spearman rank correlation test: *r* = 0.805, *P* = .001; Fig. 3B).

### 3.4 The fNIRS data analyses in PFC during WM task

**Figure 4:**
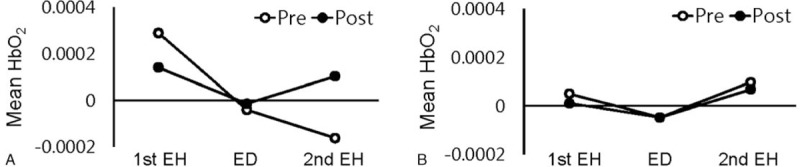
Hemodynamic changes using functional near-infrared spectroscopy pre- (session 0) and post- (session 16) NF training. The mean oxygenated hemoglobin concentration changes in the prefrontal cortex in case 3 (A) and case 4 (B) during the 1st EH before emotional distraction, ED, and 2nd EH after emotional distraction phase of the working-memory-task period. 1st EH = 1st EH delay/hold period; 2nd EH = 2nd EH delay/hold period; ED = during emotional distraction; NF = neurofeedback.

The mean accuracy on the WM task by the 5 participants with MCI improved from 18 at baseline to 40 following the NF training. We evaluated all 5 patients’ hemodynamic responses using fNIRS data; however, fNIRS data from 2 patients (cases 3 and 4) were analyzed owing to a malfunctioned computer during the WM task. Before and after NF training, compared with the rest period, the mean oxy-Hb levels in the PFC were calculated for the 1st delay/hold period (before emotional distraction), emotional distraction period, and the 2nd delay/hold period (after emotional distraction) of the WM task for case 3 (Fig. 4A) and case 4 (Fig. 4B), respectively. For case 3, concerning pre-NF training, the oxy-Hb value increased during the 1st delay/hold period and dramatically reduced during the 2nd delay/hold period after emotional distraction. After NF training, the oxy-Hb value during the 2nd delay/hold period slightly increased, which was reduced by emotional distraction before NF training. In contrast, concerning case 4, the oxy-Hb values were not affected by emotional distraction both pre- and post-NF training. Supplementary Figure 1 shows the topographical maps of oxy-Hb for case 3 (Supplementary Figure 1A) and case 4 (Supplementary Figure 1B).

### 3.5 Depression assessment

MCI is associated with a high risk for depression,^[46]^ and this association is linked to the progression to dementia.^[48]^ To reconfirm the correlation between depression and MCI and investigate the impact of NF training on depression, we assessed depression using the BDI. Mean BDI scores were 15 ± 5.61 (*P* = .144) following session 8 and 20 ± 8.46 (*P* = .893) following session 16. Compared with baseline (19.2 ± 4.27), BDI scores were not significantly different (Supplementary Figure 2).

## 4 Discussion

This study showed the effectiveness of 16 sessions of NF training applied to patients with MCI. Patients with MCI showed cognitive improvement of the MoCA-K scores and some domains of the CNSVS. The MoCA can be used to screen for MCI, and it has high sensitivity and specificity to diagnose MCI^[39]^; however, mental status examinations are imperfect tools.^[6]^ To increase reliability, we utilized the CNSVS, which is also known to be sensitive to mild cognitive dysfunction.^[42]^ In addition, we adopted hemodynamic responses in the PFC to assess brain activity during the WM task before and after NF training. WM is often distracted by emotion,^[49]^ and our fNIRS data from case 3 showed reduced mean oxy-Hb value by highly arousing and negative pictures. However, this reduction in mean oxy-Hb was recovered after NF training. Although individual differences have been identified, NF training in MCI may suggest the possibility of recovery of memory disruption by emotional challenges and improvement in cognitive function.

Several studies have reported that NF training in patients with MCI has improved memory performance. Lavy et al^[11]^ examined how NF training improves cognitive performances in patients with MCI.^[11]^ Eleven patients with MCI received NF training that comprised 10 30-minute sessions over 5 weeks and a 30-day follow-up. Memory performance using NeuroTrax^TM^ tests improved significantly following NF training, and the peak alpha frequency increased dose-dependently. Especially, memory improvement was maintained at the 30-day follow-up. Jirayucharoensak et al^[23]^ evaluated if game-based NF enhanced the cognitive performance in 54 healthy elderly participants and 65 individuals with an amnestic MCI. Game-based NF (20 sessions for 30 minutes each; 2–3 sessions per week) significantly improved spatial WM—a characteristic of amnestic MCI—and rapid visual processing. Further, Marlats et al^[22]^ published a randomized controlled trial protocol to compare the effect NF training on MCIs. Participants were assigned to an intervention group (30 sessions of sensorimotor/delta-ratio or beta/theta-ratio NF training) or a control group (psycho-pedagogical care). However, hemodynamic changes using fNIRS following NF training in patients with MCI have not been investigated. Our study revealed improved cognitive functions and hemodynamic changes in PFC following NF in patients with MCI. This indicates NF as a possible therapy for MCI.

Emotions affect cognition and behavior by enhancing or hindering them.^[29]^ The reciprocal influence between emotion and cognition is subjective, and the way individuals perceive, experience, and respond to emotionally challenging stimuli differs. Dolcos et al^[49]^ compared the neural correlates between episodic memory enhancement and WM impairing effects by emotional distraction using fMRI. The emotional pictures that disrupted WM and enhanced episodic memory were associated with increased amygdala and hippocampal activity and reduced dlPFC activity. Additionally, individual differences in the impact of emotional distraction were identified, and participants who were more susceptible to WM impairment showed greater amygdala activations and PFC reduction.

In the current study, concerning case 3, mean oxy-Hb concentration in the PFC sharply reduced following performance on the WM task with emotional distraction (Fig. 4A) before NF training. This was consistent with a previous study that showed reduced dlPFC activity by emotional distraction.^[49]^ After NF training, mean oxy-Hb concentration increased during emotional distraction and the 2nd delay/hold period while the mean oxy-Hb was less increased during the 1st delay/hold period; that is, hyperactivation to the WM task was recovered post-NF training.

Concerning our hypothesis, first, mean oxy-Hb concentration during the 1st delay/hold period of the WM task before NF training was the greatest at baseline; therefore, there may be a neural compensation effect in patients with MCI. A previous study measured hemodynamic changes by fNIRS, along with a semantic verbal fluency task, in the PFC of healthy controls and patients with MCI or AD.^[27]^ The authors showed hyperactivation in patients with MCI and hypoactivation in patients with AD as compared with controls. A neural compensation mechanism was suggested to underlie the hyperactivation, and hypoactivation was considered to reflect an inability to compensate owing to structural impairments.^[27]^ Another study showed that a neural compensation mechanism improved cognitive processing efficiency and capacity and delayed the progression from MCI to AD.^[50]^ Although to what degree hyperactivation is an indicator of a compensatory mechanism is not known, elucidating this relationship could be the key to delaying the progression from MCI to AD.

Second, mean oxy-Hb concentration during the 1st delay/hold period after NF training reduced compared with that before NF training. We suggest that the compensation effect in MCI was normalized and patients’ processing efficiency improved. In a previous study, it was reported that prefrontal activity after WM training in healthy controls decreased, indicating increased efficiency of cognitive processing.^[51]^ Another possibility is that the concentration of mean oxy-Hb would have decreased if participants were more depressed after the NF training than during the baseline. Prefrontal oxygenation, as assessed using fNIRS in patients with depression, reduces during WM tasks.^[52]^ However, we did not find differences in BDI scores after NF training as compared with those at baseline; therefore, the likelihood of an impact of depression is low.

Finally, we posit that the decrease in oxy-Hb was because of emotional distraction, which may have inhibited oxy-Hb activation. In previous studies, it was demonstrated that emotion and attention in fear conditioning had an interactive effect,^[53]^ and oxy-Hb, as measured using fNIRS in PFC during a distraction task, decreased.^[54]^ Moreover, the oxy-Hb concentration after NF training increased as compared with that before NF training during the 2nd delay/hold period. We posit that the negative effect on brain activity because of emotional distraction was restored by NF training. Although the effects of NF training on brain activity are still unclear, the current results provide some clarity.^[27]^

In contrast, unlike the above results concerning case 3, mean oxy-Hb concentration in case 4 did not change, regardless of NF training or emotional distraction (Fig. 4B). The participant in case 4 may not have expected to be susceptible to emotional distraction. Further research is needed to determine the cause of individual differences on the response to emotional challenge. Additionally, cognitive function, as evaluated with MoCA-K and CNSVS, was lower in case 4 that in case 3. Therefore, hypoactivation in case 4 may be considered to reflect an inability to compensate.

## 5 Conclusion

In conclusion, NF training for MCI is likely to improve cognitive function. This study also provides insight into possible brain-activity mechanisms when patients with MCI complete WM tasks. Reduced hemodynamic responses by emotional distraction were restored by NF training, indicting the utility of NF training in patients with MCI, which may be used as a non-pharmacological therapeutic tool for MCI.

Despite these strengths, some limitations of the current study should also be noted. Our study is an open-label trial with a small sample size. Second, there was no long-term follow-up to demonstrate maintained cognitive improvement post-NF training. Further, only single-channel EEG recording was used. Further studies are needed, such as large-scale randomized controlled trials with follow-up, to confirm the beneficial effects and brain-activity mechanisms underlying NF training in individuals with MCI.

## Acknowledgments

The authors thank Professor Inchul Jung (Dunsan Korean Medicine Hospital, Daejeon University) for his help.

## Author contributions

**Conceptualization:** Jung-Hee Jang, Jieun Kim, Gunhyuk Park, Jun-Hwan Lee, Horyong Yoo.

**Data curation:** Siyeon Kim.

**Funding acquisition:** Jun-Hwan Lee, Horyong Yoo.

**Methodology:** Jung-Hee Jang, Jieun Kim, Gunhyuk Park, Eun-Sun Jung, Ji-yun Cha Cha, Chan-young Kim.

**Supervision:** Jun-Hwan Lee, Horyong Yoo.

**Writing – original draft:** Jung-Hee Jang.

**Writing – review & editing:** Jieun Kim.

## Supplementary Material

Supplemental Digital Content

## Supplementary Material

Supplemental Digital Content
