# Supplementary material for: Beta wave enhancement neurofeedback improves cognitive functions in patients with mild cognitive impairment: A preliminary pilot study
Source: Medicine (Baltimore). 2019 Dec 16;98(50):e18357. doi: 10.1097/MD.0000000000018357 (PMC6922450; doi:10.1097/MD.0000000000018357)
Supplement: Supplemental Digital Content [file medi-98-e18357-s001.pptx]

## Slide 1
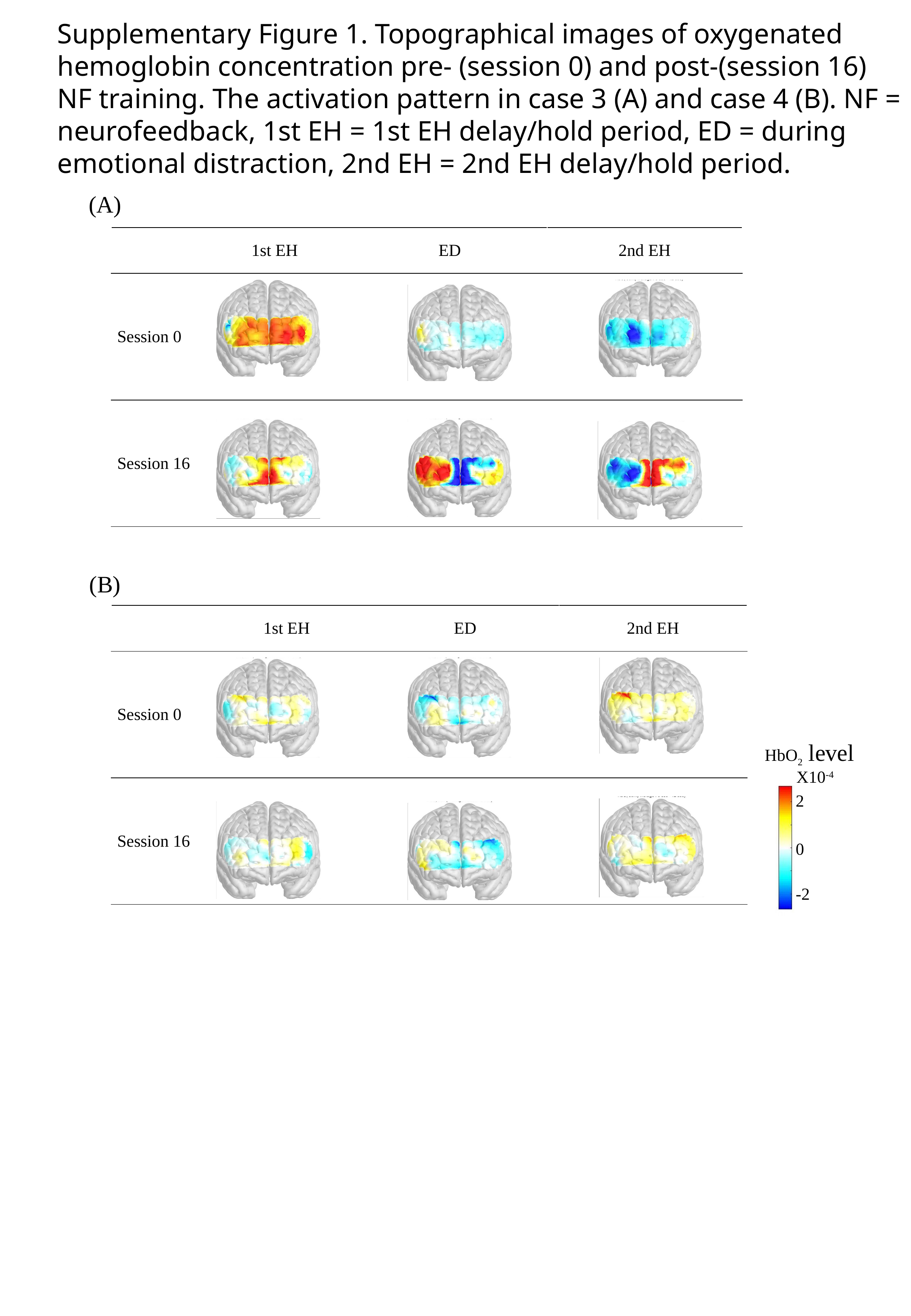

Supplementary Figure 1. Topographical images of oxygenated hemoglobin concentration pre- (session 0) and post-(session 16) NF training. The activation pattern in case 3 (A) and case 4 (B). NF = neurofeedback, 1st EH = 1st EH delay/hold period, ED = during emotional distraction, 2nd EH = 2nd EH delay/hold period.
(A)
| | 1st EH | ED | 2nd EH |
| --- | --- | --- | --- |
| Session 0 | | | |
| Session 16 | | | |
(B)
| | 1st EH | ED | 2nd EH |
| --- | --- | --- | --- |
| Session 0 | | | |
| Session 16 | | | |
HbO2 level
X10-4
2
0
-2
