# Supplementary material for: Beta wave enhancement neurofeedback improves cognitive functions in patients with mild cognitive impairment: A preliminary pilot study
Source: Medicine (Baltimore). 2019 Dec 16;98(50):e18357. doi: 10.1097/MD.0000000000018357 (PMC6922450; doi:10.1097/MD.0000000000018357)
Supplement: Supplemental Digital Content [file medi-98-e18357-s002.pptx]

## Slide 1
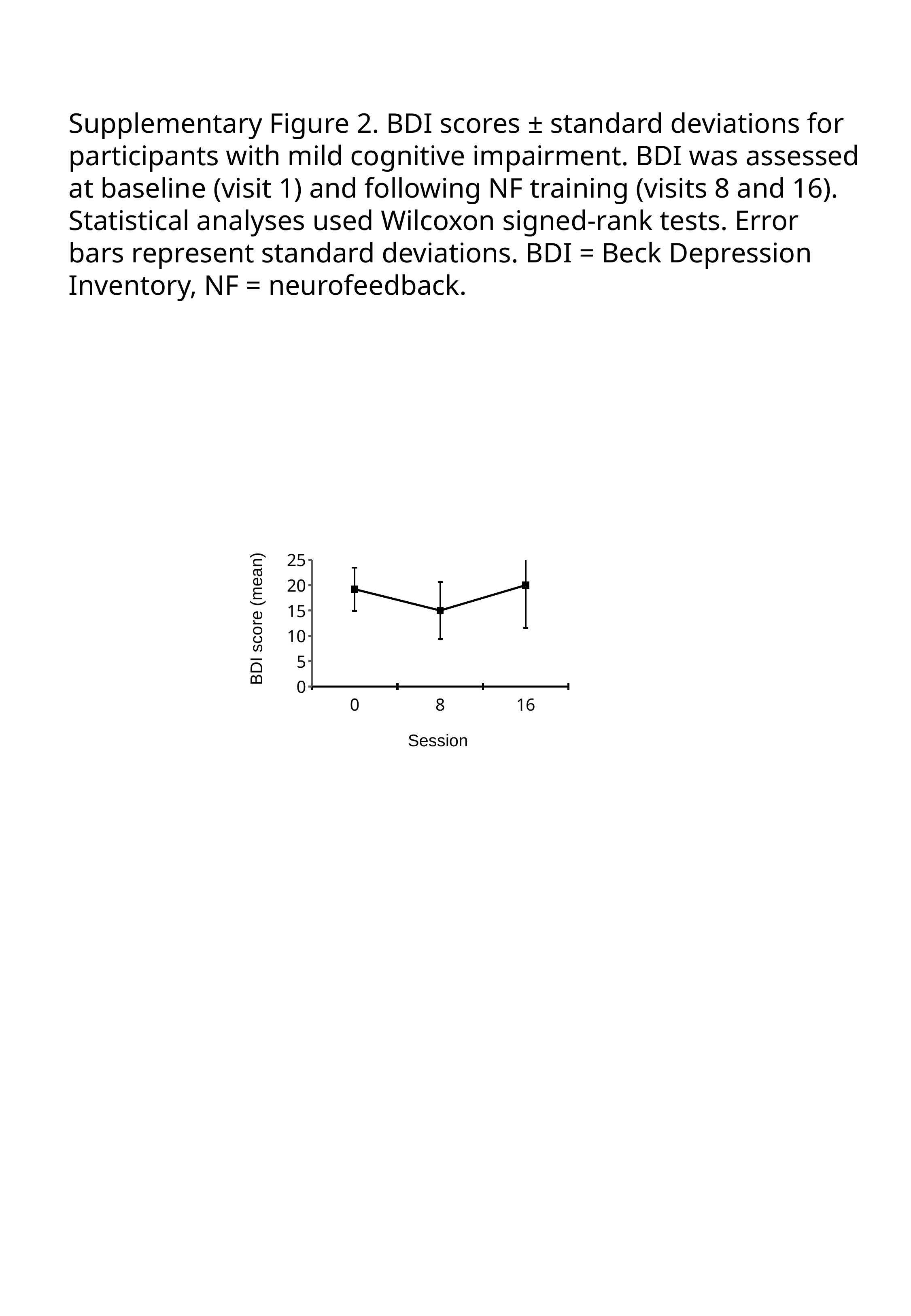

Supplementary Figure 2. BDI scores ± standard deviations for participants with mild cognitive impairment. BDI was assessed at baseline (visit 1) and following NF training (visits 8 and 16). Statistical analyses used Wilcoxon signed-rank tests. Error bars represent standard deviations. BDI = Beck Depression Inventory, NF = neurofeedback.
### Chart
| Category | |
|---|---|
| 0 | 19.2 |
| 8 | 15.0 |
| 16 | 20.0 |BDI score (mean)
Session
